# Pre-school-based behaviour change intervention to increase physical activity levels amongst young children: a feasibility cluster randomised controlled trial

**DOI:** 10.3389/fpubh.2024.1379582

**Published:** 2024-05-02

**Authors:** Mosfer A. Al-walah, Michael Donnelly, Adel A. Alhusaini, Neil Heron

**Affiliations:** ^1^Centre for Public Health, School of Medicine, Dentistry and Biomedical Sciences, Queen’s University Belfast, Belfast, United Kingdom; ^2^Department of Physical Therapy, College of Applied Medical Sciences, Taif University, Taif, Saudi Arabia; ^3^Department of Rehabilitation Sciences, College of Applied Medical Sciences, King Saud University, Riyadh, Saudi Arabia; ^4^School of Medicine, Keele University, Newcastle-Under-Lyme, Staffordshire, United Kingdom

**Keywords:** feasibility, physical activity intervention, sedentary behaviour, childhood obesity, young children, prevention, Saudi Arabia

## Abstract

**Background:**

A significant rise in childhood obesity worldwide over the past three decades highlights the urgent need for early interventions, especially in preschools as key settings for child development. This study aimed to assess the feasibility and fidelity of a randomised controlled trial of “I’m an Active Hero” (IAAH), a theory- and evidence-based multi-component behaviour change intervention targeting physical activity and sedentary behaviour amongst preschool-aged children.

**Methods:**

Two preschools in Taif city, Saudi Arabia were randomly assigned to either the intervention (*n* = 3 classrooms) or the usual curriculum control group (*n* = 3 classrooms). The intervention ran for 10 weeks from February to April 2023 and consisted of teacher-led physical activity and sedentary behaviour sessions in preschools, with an additional interactive home component. Primary outcome measures included intervention fidelity, recruitment rates, attrition rates, and compliance with trial procedures. Secondary outcomes included body mass index (BMI), objectively measured physical activity, and sedentary time via the ActiGraph GT3X accelerometer. Outcomes were measured at baseline and at 10 weeks in both study arms.

**Results:**

The preschool intervention component had high fidelity (93.3%), but the home component fidelity was lower (74%). A cluster-level recruitment rate of 12% (13/112 centres) was attained, whilst the individual-level recruitment rate stood at 36% (52/143 children, mean age of 4.16 years; 23 girls). Attrition was 10%. Compliance varied with 90% for BMI, 71% for accelerometery, and 45% for questionnaires. The intervention group showed small decreases in BMI, slight increases in physical activity, and decreases in sedentary time at follow-up compared to the control group. Parents, facilitators, and assistant teachers considered the intervention to be feasible and beneficial.

**Conclusion:**

The IAAH intervention was feasible to implement in Saudi Arabian preschools. Facilitators showed high fidelity in delivering it. However, preliminary data did not demonstrate effectiveness. A more comprehensive evaluation across a broader population is warranted. The intervention could be revised to optimise recruitment, compliance, and fidelity of the home-based component. Successful elements from this pilot should be retained whilst adaptations to implementation are made to strengthen key areas.

**Clinical trial registration**: ClinicalTrials.gov, NCT05754359.

## 1 Introduction

Physical activity (PA) constitutes a significant behavioural factor which is intricately associated with obesity during early childhood (1, 2). An expanding body of research has underscored the vital role of PA from birth to age 5 in promoting positive health outcomes (3, 4) including enhanced bone density, cardiovascular health, body composition, and cognitive and motor development (4, 5). Furthermore, the early childhood phase is a crucial period for establishing enduring tendencies toward physical activity, as patterns formed during this time can persist into middle childhood (6, 7) and early adulthood (8).

Current PA guidelines recommend that preschoolers accumulate at least 180 min per day of light, moderate to vigorous intensity PA (LMVPA) (9–11). However, recent evidence has suggested a concerning trend whereby a substantial number of preschoolers worldwide are not meeting these recommended PA guidelines. According to research, between 62 and 90% of young children do not meet the recommended level of PA for optimal health benefits (12, 13). Furthermore, studies from the United States have indicated that more than half of preschool children do not fulfil recommended PA standards (14). Compounding this issue, most preschoolers spend the majority (79%) of their day engaged in sedentary behaviours (SB) (15–18).

Childhood obesity represents one of the most pressing global public health challenges, as both developed and developing nations grapple with excessive population weight gain (19). Recent estimates indicate that over 40 million children under 5 years of age worldwide are affected by overweight or obesity (20), and without effective preventative measures, the global prevalence of childhood obesity is projected to increase exponentially to 91 million by 2025 (21). Obese children face increased risks of developing hypertension, cardiovascular disease, and diabetes (22). They are also more likely to remain obese into adulthood (23, 24).

Saudi Arabia faces a concerning prevalence of obesity across all ages and genders, including preschool populations (25, 26). From 1980 to 2013, Saudi Arabia experienced one of the most pronounced increases in obesity rates globally (25, 27), leading to its current ranking amongst the top 10 countries with the highest proportion of overweight/obese individuals (28). This worrying trajectory persists (25). Research on early indicators of overweight and obesity in Saudi preschool children (aged 2–6 years) reveals an alarming rate of overweight and obesity (29). Therefore, developing etiological insights into determinants of early childhood obesity within the Saudi cultural context, compared to global benchmarks, is an urgent public health imperative (29, 30). Early intervention programmes to prevent childhood obesity are critically needed as, otherwise, the long-term health and economic impacts could be very significant (31, 32).

Globally, most preschool-aged children are enrolled in early childcare programmes in which they spend a majority of their day (33). With preschool attendance exceeding 8 h per day for most children, these settings have become the primary setting of care and education during early childhood. Consequently, preschools have garnered increasing attention as potentially efficacious venues for improving PA levels amongst this population (34, 35). Multicomponent interventions which target PA and SB, both in the preschool and home environment, tend to show the most promise with regard to improving energy-balance-related behaviours and mitigating unhealthy weight gain trajectories in early childhood (36, 37).

Over the past decade, numerous PA interventions have been implemented and empirically evaluated in preschools in an effort to increase young children’s PA levels and address the global childhood obesity epidemic (34, 37–40). However, intervention effects have proven largely heterogeneous thus far, underscoring the need for the continued optimisation and refinement of preschool-based PA promotion. Most experimental interventions to date have taken place within the home (17, 41) or childcare-based settings (39, 42). Syntheses of the literature have indicated intervention components characterised by structured PA opportunities, parental involvement, expert delivery agents, and grounding in behavioural theory, as representing critical determinants of intervention success (37). However, a limitation of the extant literature is the geographical concentration of studies largely within developed Western nations such as the United States, Canada, Australia, and Western Europe. Very little research in this area has been carried out within other global contexts such as the Middle East. Given previous findings indicating a critical research gap and an urgent need for interventions targeting obesity-related behaviours amongst preschool children in Saudi Arabia, there is still a lack of preschool interventions promoting healthy PA. No intervention programme for childhood obesity has been implemented or administered in this population to date.

To address this research gap and align with the established need for a systematic approach to intervention development, we executed a multi-phase process. The objective was to identify an optimal delivery mode, components, and content for a novel intervention, “I’m an Active Hero” (IAAH), designed to address PA and reduce SB in young children. This preschool-based behaviour change intervention, incorporating family engagement, followed a systematic development process in accordance with the Medical Research Council (MRC) Framework for the Development and Evaluation of Complex Interventions (43, 44). This framework outlines key steps, including development, feasibility, evaluation, and implementation. The initial phase involved a comprehensive systematic review to identify behaviour change techniques associated with increased PA in preschoolers (45). Subsequently, qualitative research was undertaken, incorporating input from key stakeholders (including principals, teachers and parents) through focus groups and interviews (46). This approach aimed to establish priorities and objectives tailored to our target demographic.

The Socio-ecological model (SEM) (47) and social cognitive theory (SCT) (48) served as our theoretical foundation, elucidating the expected mechanisms of behaviour change. The preschool-based IAAH intervention, complete with family involvement, was meticulously designed, following the Template for Intervention Description and Replication framework (49) and methodically mapped to specific behaviour change techniques (50). Informed by evidence suggesting superior performance, we opted for face-to-face delivery and supervision as the most favourable implementation strategies (51, 52).

However, the MRC recommends conducting a feasibility study before launching full-scale effectiveness trials, considering it a vital step in developing and evaluating interventions (53). This phase offers significant advantages, chiefly identifying potential limitations in the study design, intervention delivery, or components that could undermine its benefits for the target population. Addressing such issues at an early stage avoids expending significant resources on a fully powered trial when fundamental flaws exist that preclude intended outcomes. Beyond conserving often scarce resources, optimising protocols and methods at this stage also enhances subsequent randomised trials’ integrity and impact.

Moreover, within a context of limited public health resources, confirming feasibility brings economic benefits by ensuring that investments in scaling avoid funding an expensive yet ineffective programme. Therefore, the aim of this study was to test the feasibility of a cluster randomised controlled trial (RCT) of the IAAH programme to inform amendments prior to conducting a larger scale evaluation, which is likely to be useful for policy and add to the existing body of knowledge in this field. This will highlight a variety of aspects that emphasise the importance of a comprehensive intervention programme that would serve as a basis for future obesity-related interventions. Notably, this constitutes pioneering research in Saudi Arabia as it is the first such study to be conducted in this context.

## 2 Methods

### 2.1 Study design

This study adhered to the guidelines outlined in the CONSORT statement’s extension for randomised pilot and feasibility trials (54). The trial utilised a cluster-randomised controlled design, with preschools serving as the units of randomisation and individual children as the units of analysis. The study involved two preschools, each with three classrooms, which were randomly assigned to either the intervention or control condition. As this was a feasibility study, no formal sample size calculation was performed. The participating preschools were matched based on key characteristics such as size and demographics, eliminating the need for pre-randomisation matching procedures. To assign preschools to study conditions, an impartial researcher randomly selected one of two opaque envelopes, prepared by a separate team member, containing the names of the participating schools. One envelope was allocated to the control group, and the other was assigned to the intervention. Data collection occurred at two time points, baseline and post-intervention, spanning February to April 2023. This study was approved by the Saudi Arabian Ministry of Health’s Research and Studies Department (IRB Registration Number with KACST, KSA: HAP-02-T-067).

### 2.2 Setting, sampling, and participants

In the context of Saudi Arabia, a substantial proportion of young children enrol in both public and private preschools. This study was carefully designed to take place within preschools situated in the city of Taif, Saudi Arabia. These preschools are officially registered with the Ministry of Education and adhere to national curriculum guidelines. To initiate the study, a representative from Taif City Council contacted a convenience sample of all locally operated kindergartens within the geographic boundaries of Taif City through electronic communication to assess interest in participating (*n* = 112). Of these schools, 13 preschools expressed their willingness to participate in the study. Through a considered selection process, two preschools with similar demographics were chosen based on their size, socioeconomic status (SES), and demographic composition.

The study director personally visited the principals of the selected preschools, providing them with comprehensive information sheets and consent forms. These documents were subsequently distributed by principals to parents/caregivers of all 3–5-year-old children at their preschools. Exclusion criteria were applied to children with significant health issues that could impede participation or those lacking parental consent. The intervention was then delivered to all eligible 3–5-year-old children in the intervention preschool.

### 2.3 Intervention

#### 2.3.1 The IAAH intervention programme

The IAAH intervention is a 10-week, preschool-based behaviour change programme aimed at increasing PA amongst 3–5-year-old children. A 10-week duration was selected to align with the local preschool calendar and allow for adequate time to assess short-term experimental effects (55). This programme was delivered by preschool teachers who had undergone two preparatory sessions directed by the lead researcher. This face-to-face method of delivery was adopted based on prior research suggesting its effectiveness (37). Details of the intervention’s development will be provided in a separate publication. Briefly, the IAAH programme focused on two key behaviours related to energy balance: PA and sedentary time. The programme involved materials used both in the preschool and at home.

##### 2.3.1.1 Intervention materials to promote PA in the preschool

###### 2.3.1.1.1 Setting environmental changes in the preschool

This emphasised “unstructured PA”—the spontaneous PA that children engage in during recess with minimal teacher intervention. A classroom activities guide provided examples of how to modify the classroom to develop a more PA-conducive, friendly, and fun environment.

###### 2.3.1.1.2 Structured PA sessions

Ten physical education sessions were crafted to guide teachers on organised PA activities for children. Apart from unstructured PA, children were offered two structured PA sessions each week lasting between 45 and 60 min. These sessions aimed to bolster their movement skills and elevate their PA. They encompassed playful exercises targeting endurance, coordination, speed, strength, and flexibility.

###### 2.3.1.1.3 Classroom movement breaks

These were brief PA interludes designed to punctuate prolonged sitting periods. Over 10 weeks, trained teachers integrated 3-5-min movement breaks into their daily routines, accumulating 15 min of PA on four school days per week. The breaks were tailored for classrooms and designed for limited-space environments. They were flexible and easily incorporated into the preschool day with minimal disruption. They included interactive, fun, and non-competitive activities aimed at seamlessly integrating PA into academic lessons. Teachers received classroom guides outlining activities to deliver throughout the day and week. They were encouraged to deliver a total of 1 h of activities per week, gradually introducing more advanced options as the intervention progressed. Educators also received resources supporting PA break delivery.

##### 2.3.1.2 Intervention materials to promote PA at home

Educational materials were provided to parents/caregivers whilst the intervention was implemented at the preschools. These included three newsletters, three tip cards, and two posters promoting PA and reducing SB.

The newsletters and tip cards offered practical, easy-to-understand advice for families from all socioeconomic backgrounds participating in the IAAH intervention. They explained the importance of daily PA for preschoolers and provided suggestions for integrating movement into family routines, including simple ideas for everyday life, active weekend excursions, and being physically active role models. Posters displayed brief messages emphasising the implementing of PA into daily family life (e.g., “Keep moving!” “The car is a ‘movement killer’!” “Come to kindergarten actively!”).

Compared to previous studies (56, 57) that used passive techniques like tip cards and newsletters, the IAAH used interactive games and activities requiring active involvement from both parents and children. Families also received “No TV Day” challenges to potentially decrease sedentary time and increase active family time.

To ensure effective implementation, early years practitioners in the intervention group underwent two distinct 3-h training sessions led by the primary researcher. The first session took place before the intervention’s commencement, whilst the second occurred 5 weeks later. During the initial session, educators were briefed on the study’s background, objectives, and details, with a focus on the IAAH programme. They were also reminded of the significance of embodying a role model for cultivating a healthy, active lifestyle. This session provided an opportunity for preschool teachers to clarify their queries. Additionally, during this training session (i.e., before the start of the intervention), educators received the IAAH materials including a teacher’s guide, classroom activity guides, newsletters, tip cards, and posters. The teacher’s guide provided background information on defining PA and underscored the importance of increasing preschoolers’ PA levels to establish healthy behaviours. The classroom activity guide for PA included three themes: (1) setting classroom environmental changes (retained all year), (2) children performing PA during structured sessions (implemented for 10 weeks), and (3) movement break classroom activities (also implemented for 10 weeks). The training also explained the home component and how to deliver it to parents. The subsequent session, scheduled at the midpoint, was designed to share experiences and reaffirm motivation and enthusiasm.

### 2.4 Procedures and outcomes

Participants were evaluated at two timepoints by two appraisers: one researcher (MA) and a field assistant. Both appraisers went through training for the measurement procedures. Initial assessments were conducted in February 2023, followed by a second round of measurements 10 weeks later. To prepare children and address any potential issues during data collection, an early childhood educator from each preschool was present. Whilst parental permission was obtained for all participating children, child assent was also obtained before measurements began. Children unwilling to take part in specific data collection procedures were not obliged to do so. Since the main focus of this study was examining the feasibility of the intervention and trial, key outcomes of interest included recruitment rates, attrition rates, implementation fidelity, and compliance with data collection. Additionally, several secondary outcomes, as described below, were also assessed.

### 2.5 Trial feasibility, recruitment and retention

These records encompassed crucial information pertaining to recruitment, which included initial outreach to potential schools and participants, details concerning individuals excluded from the study, those who expressed a willingness to participate further, as well as retention data, encompassing the number of participants who withdrew, were lost to follow-up, or provided data. Measurement sessions were a fundamental aspect of this study, and they were exclusively conducted at participating schools. These sessions were strategically scheduled at two key time points: the baseline assessment and a follow-up evaluation after a span of 10 weeks.

### 2.6 Implementation fidelity

Implementation fidelity refers to how much an intervention is carried out as planned by its developers (58). Fidelity was evaluated in both preschool and home settings using the following approaches:

#### 2.6.1 Preschool component

To evaluate the implementation fidelity of the preschool programme, practitioners were provided with monthly logbooks which were based on the model of Saunders et al. (59, 60). These logbooks were utilised to document the execution of the programme throughout the intervention period. Using 5-point Likert scales, practitioners rated their monthly implementation of key programme components including modifications to the classroom layout, student engagement in targeted health behaviours, and the integration of health concepts into classroom activities and routines. This logbook methodology allowed for the systematic documentation of practitioner adherence to prescribed programme components on a month-to-month basis. All intervention teachers were asked to complete the log sheet to record frequency (number of times per day they implemented the intervention) and duration (length of each intervention).

#### 2.6.2 Home component

To evaluate the reach of the home-based component of the intervention, practitioners documented the number of eligible children who received activity packs each month. Post-intervention, parents/caregivers completed a questionnaire (see Supplementary File S1) to assess their receipt of and engagement with the home materials. The questionnaire utilised binary yes/no response options and 5-point Likert scales. Questions were designed to determine if parents/caregivers obtained the intervention materials and used them at home with their child.

### 2.7 Attendance at sessions and intervention harms

Participant attendance at intervention sessions was documented at each session by the facilitator. The facilitator was also tasked with recording any accidents or injuries occurring as a result of the intervention.

### 2.8 Secondary outcome measures

#### 2.8.1 Body mass index

Anthropometric measurements were conducted by a trained researcher (MA) under standardised conditions to ensure accuracy and reliability. Children were measured wearing light clothing and barefoot in a private room with 3–4 children present at a time. Height was measured to the nearest 0.1 cm using a stadiometer (Marsden, UK) and weight was measured to the nearest 0.1 kg on an electronic scale (Tanita, Amsterdam, Netherlands). Two measurements were taken for both height and weight and the average for each was calculated.

BMI were derived from the weight and height data using standardised anthropometric measurement techniques (61). This involved utilising age- and sex-specific reference data from the UK90 growth charts for children aged ≥4 years (62), as well as the World Health Organization growth charts for 3-year-olds (63). Based on BMI percentiles, children were classified as being of normal weight (<85th percentile), overweight (≥85th to <95th percentile), or obese (≥95th percentile) (64).

#### 2.8.2 Objectively measured PA

PA was objectively measured using the ActiGraph GT3x accelerometer (ActiGraph, Pensacola, Florida, USA). The ActiGraph GT3x is a small, wearable device that attaches to the front of the mid-thigh and measures postural information. The device categorises activity into sitting/lying, standing, and moving/stepping (65). Once attached, the ActiGraph GT3x can be worn continuously for 7–10 days (66). Preschool educators fitted participants with the ActiGraph GT3x under researcher instruction. Parents were asked to place an ActiGraph GT3X accelerometer on a waistbelt on their child’s right front hip. Participants were asked to wear the monitor during all waking hours for four consecutive days, including two weekend days (i.e., Wednesday to Sunday) each at baseline and 10 weeks, only removing it when the monitor would get completely wet. A motivational sticker chart was provided to encourage adherence to accelerometer wear. To assess PA patterns, the preschool day was divided into preschool (8:00 a.m.–2:30 p.m.) and after-school/evening hours (2:31 p.m.–10:00 p.m.). For inclusion in the analysis, participants were required to have a minimum of 8 h of accelerometer data per day, covering at least one weekend and two weekdays at each time point. The selection of PA outcome variables, cutoff points, and validation criteria were guided by precedent set in a prior study involving preschool children (11, 67, 68).

#### 2.8.3 Objectively measured SB

Sedentary time during waking hours was assessed using the ActiGraph GT3X accelerometer following the same procedures used for PA measurement (69). Periods of nighttime sleep were differentiated from waking SB by examining the raw accelerometer data files to identify extended periods without significant changes in axis of movement (indicating a transition from sitting/lying to standing), which denote times when the participant was asleep (69).

### 2.9 Analysis

To evaluate implementation fidelity in this study, we adapted scoring systems used by previous studies (59, 60, 70) to code teachers’ logbook and questionnaire responses that indicated the level of implementation. For dichotomous items, a positive response (yes) received a code of 1, whilst a negative response (no) was coded as 0. For Likert scale items, a response of 4 (agree/often) or 5 (strongly agree/always) was coded as 1, whilst all other responses (1-3) were coded as 0. Total implementation fidelity scores of 18 and 12 were revealed for teachers and parents, respectively, which were based on the model of Saunders et al. (59, 60).

To determine and categorise participant weight status from height and weight measurements, data was entered into the LMS Growth Excel add-in to generate percentile scores (71).

Accelerometer raw count data was processed using ActiLife version 6 software (ActiGraph, Pensacola, Florida, USA) and integrated into 15 s epochs (72). Non-wear time was defined as ≥20 consecutive minutes of zero counts. A valid wear-time was ≥8 h on any 3 days. Pate cut points (73) were used to estimate daily moderate-to-vigorous PA (MVP), total and light PA, steps, and sedentary time.

As this was a feasibility study with a small sample size, inferential statistics and effectiveness testing were not recommended (74, 75). Instead, descriptive statistics were used to assess feasibility parameters including fidelity of implementation, recruitment, retention, and attrition rates, presented as proportions. High, medium, and low fidelity were classified as overall implementation scores of ≥60%, ≥50 to <60%, and < 50%, respectively, following the methodology of similar studies (58). For secondary outcomes, the study presented means ± standard deviations, along with the mean change from baseline to follow-up and 95% confidence intervals where appropriate.

## 3 Results

### 3.1 Feasibility of trial recruitment and retention

**Figure 1:**
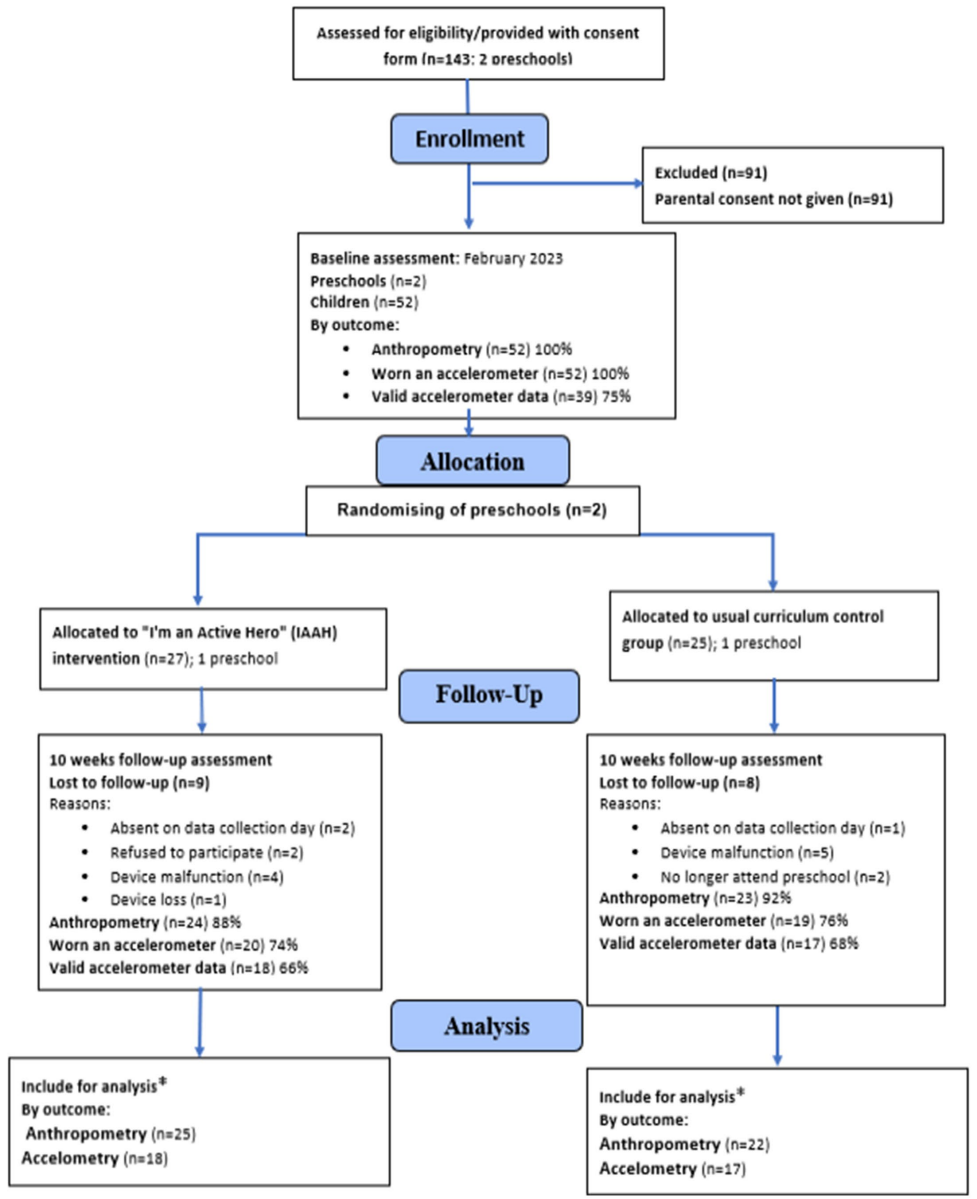
CONSORT flow diagram detailing trial recruitment and retention for the IAAH intervention. *Only participants that provided both baseline and post-intervention data were included within subsequent analyses.

The study received responses from thirteen out of 112 preschools, indicating a cluster-level response rate of 12%. Amongst the 143 consent forms distributed, 52 children (mean age 4.17 ± 0.145 years; 23/44% girls) obtained parental consent and completed the baseline assessment, resulting in an individual-level recruitment rate of 36% before preschools were randomised to the IAAH intervention arm (1 centre; *n* = 27; 13 girls) or the usual curriculum control arm (1 centre; *n* = 25; 10 girls). A CONSORT flow diagram illustrating the study’s progression is presented in Figure 1.

Independent t-tests showed no significant baseline differences between the intervention (*n* = 27) and control (*n* = 25) groups in age (intervention: 4.18 ± 0.42 years; control: 4.16 ± 0.49 years; *p* > 0.05) or mean BMI (intervention: 16.38 (2.14) kg/m2; control: 16.58 (1.89) kg/m2; *p* > 0.05). Participant characteristics at baseline are presented in Table 1.

**Table 1:** Baseline descriptive statistics of participants in the intervention and control groups.

Characteristics	Control (*n* = 25)	Intervention (*n* = 27)	*p*-value	All (*n* = 52)
Age (years) (mean SD)	4.16 (0.49)	4.18 (0.42)	0.90	4.17 (0.46)
*N* (%) girls	11 (44)	12 (44.4)	0.97	23 (44.2)
Height cm (mean SD)	100.78 (5.17)	101.45 (5.26)	0.64	101.11 (5.2)
Weight kg (mean SD)	16.86 (2.52)	17.01 (2.86)	0.86	16.93 (2.7)
Body mass index (BMI) Kg/m^2^ (mean SD)	16.58 (1.89)	16.38 (2.14)	0.75	16.48 (2.02)
BMI category *n* (%)				
Obese	3 (11.1%)	2 (8%)	0.10	5 (9.6%)
Overweight	4 (14.8%)	5 (20%)	0.73	9 (17.30%)
Normal	15 (55.6%)	17 (68%)	0.72	32 (61.5%)
Under weight	2 (7.4%)	1 (4%)	0.65	3 (5.7%)

The accelerometer-based PA data showed no significant observed between-group differences at baseline for any PA variables including vigorous PA, moderate-to-vigorous PA (MVPA), and sedentary time. Over 70% of baseline activities were sedentary in both groups. Additionally, 17.3% of participants were considered overweight as per the BMI. Descriptive PA data at baseline (Time 1) and post-intervention (Time 2) are presented in Table 2.

**Table 2:** Summary of results for participants that completed PA measurement at baseline and follow-up.

Variable	*n*	Baseline, M (SD)	*n*	10 weeks M (SD)	Adjusted within-group change from baseline to 10 weeks			At 10 weeks (post-intervention) comparison between groups		
					*M*	(95% CI)^a^	*p* value	*M*	(95% CI)^b^	*p* value
Total daily SB (min)										
Control	19	398.11 (71.75)	17	407.01 (66.45)	8.9	(65.9-,37.9)	0.571			
Intervention	20	406.33 (93.5)	18	388.26 (69.7)	−18.07	(−45.5, 66.9)	0.694			
Group X time interaction								−18.8	(−67.4, −29.9)	0.085
Light PA (min/day)										
Control	19	113.09 (22.33)	17	118.5 (31.87)	−6.54	(−23.8-,10.72)	0.434			
Intervention	20	112.975 (39.4)	18	115.83 (27.7)	−2.85	(−29.3, −23.63)	0.623			
Group X time interaction								−2.7	(−20-,14.07)	0.076
Mod PA (min/day)										
Control	19	36.54 (13.52)	17	35.11 (9.57)	2.11	(−7.04-11.27)	0.631			
Intervention	20	35.56 (11.8)	18	40.49 (10.5)	−5.98	(−14.06–2.09)	0.137			
Group X time interaction								−2.7	(−20–14.07)	0.0.076
MVPA (min/day)										
Control	19	48.01 (13.34)	17	44.49 (13.62)	−4.04	(−6.11–14.20)	0.411			
Intervention	20	47.25 (13.7)	18	54.41 (16.9)	8.01	(−45.5 66.9)	0.147			
Group X time interaction								9.91	(−0.62–20.5)	0.064
Total daily PA (CPM)										
Control	19	161.10 (28.23)	17	163.001 (39.43)	−2.49	(−25.63–20.36)	0.820			
Intervention	20	159.79 (44.79)	18	170.24 (35.89)	−10.9	(−42.9–21.2)	0.485			
Group X time interaction								7.23	(−15.06–29.5)	0.051
Total daily steps (count)										
Control	19	8826.88 (3165)	17	8985.19 (2695)	−187.1	(−2,469–2094)	0.864			
Intervention	20	8965.204 (3030)	18	10130.2 (2775)	−1,223	(−2,746–298.5)	0.108			
Group X time interaction								1,144	(−899–3,188)	0.067
Wear time (min/day)										
Control	19	559.22 (73.06)	17	570.01 (73.31)						
Intervention	20	566.12 (68.257)	18	558.5 (59.23)						
Group X time interaction										0.061

Values are mean SD. SB, sedentary behaviours; PA, physical activity; CPM, counts per minute; Min, minute; Mod, moderate; MVPA, moderate-vigorous PA. ^a^All variables were adjusted for their respective baseline value, age, wear time and gender. ^b^The adjusted delta differences between groups are significant at the 0.05 level.

The assessment of initial intervention effects on PA variables during preschool hours, as depicted in Table 3, did not demonstrate significant differences between the evaluated groups. However, we noted a promising trend toward increased MVPA and decreased sedentary time in the intervention group, as evidenced by *p*-values of 0.058 and 0.063, respectively. These findings suggest the potential effectiveness of our intervention in promoting positive changes in these variables amongst preschool children. The observed trends underscore the potential of structured interventions to significantly impact PA levels within educational settings, despite the inherent challenges of modifying activity behaviours amongst this young demographic. Nonetheless, the small sample size and short duration of the intervention may have limited our ability to detect statistically significant differences in PA levels. This indicates the need for further research with longer durations and larger sample sizes to definitively ascertain the intervention’s impact.

**Table 3:** Physical activity variables during preschool day.

Variable	Intervention (*n* 20)		Control *n* (19)		*p* value
	Baseline week 10		Baseline week 10		
During preschool PA (% time spent)					
Sedentary PA	70.73 (4.2)	68.01 (4)	70.56 (6.3)	72.07 (3.7)	0.063
Light PA	20.91 (3.3)	21.94 (3.8)	20.49 (3.6)	21.07 (2.1)	0.082
MVPA	8.53 (3.1)	9.85 (2.9)	8.74 (3.1)	8.86 (2.1)	0.058
After-school/evening (% time spent)					
Sedentary PA	70.11 (5.2)	69.42 (4.2)	70.15 (6.6)	71.34 (3.8)	0.082
Light PA	20.72 (3.4)	21.41 (3.7)	20.31 (3.7)	20.96 (2.3)	0.093
MVPA	8.24 (3.2)	8.65 (2.7)	8.31 (3.2)	8.73 (2.2)	0.124

All values are the mean and standard deviation.

### 3.2 Intervention fidelity

The intervention preschool submitted complete activity logbooks for the 10-week study. Overall implementation fidelity across the intervention preschool was high at 93.3% (Table 4). Intervention components related to PA were implemented with higher fidelity compared to SB components based on the logbook data. The post-intervention survey indicated a 74% overall implementation score for the home-based intervention component. Table 4 provides detailed preschool implementation fidelity scores from the practitioner logbook data.

**Table 4:** Preschool implementation fidelity score logbook items and responses.

Component	Logbook question	Scoring and results (% coded as 1 over the 3 months)	
		PS (%)	(%) (Fidelity score)
Preschool environment	Was equipment and space appropriately arranged for PA sessions every day of the week?*	100	100 (high)
	Was the classroom appropriately arranged for movement breaks every day of the week?*	89	89 (high)
	Were any movement corners set up and made available to the children?*	100	100 (high)
Children performing the health behaviours	How much time did you devote to PA sessions on an average weekly basis this month?^+^	98	98 (high)
Classroom experiences	Did you implement the classroom experiences for PA as described in the manual?*	100	100 (high)
	Did you devote on average at least 1 h per week to the classroom activities for PA as described in the manual?*	97	97 (high)
	Did you devote on average at least 1 h per week to the classroom activities for SB as described in the manual?*	86	86 (high)
	Which classroom activity (ies) regarding PA did you implement this month?^+^	98	98 (high)
	Which classroom activity (ies) regarding SB did you implement this month?^+^	85	85 (high)
Delivery of home materials and engagement with parents	Did you provide parents with the pre-prepared home activity packs when these were delivered to the nursery?*	100	100 (high)
	Estimate the number of parents to whom you directly delivered programme materials, if you did^+^ (total 29 children)	31	31 (low)
	Estimate the number of parents with whom you spent time explaining the purpose of the material and encouraging them to follow the recommendations of the material^+^ (total 29 children)	100	100 (low)
	Total aggregate scores (% responses coded as 1. Total available points = 12)	93.3	Overall score = 93.3

This form was repeated three times, once for each month the intervention was delivered. *Scoring determined by 5-point scale, “1 = never, 2 = not often, 3 = sometimes, 4 = often, 5 = always,” ≥ 4 = 1; ≤ 3 = 0. ^+^Scoring determined by a “yes/no” response or a numerical response. Yes = 1; no = 0. Numerical responses equate to ≥ 60% = 1; <60% = 0. PS = preschool.

### 3.3 Attendance at sessions and adverse events

No intervention-related adverse events, accidents, or injuries were reported by the IAAH facilitators. Facilitators indicated that intervention sessions were well-attended on a consistent basis. Attendance was generally high, with approximately 95% of participants attending each session according to facilitator and staff records. No barriers to attendance or participation were identified during the 10-week intervention period.

### 3.4 Participation in outcome measures

#### 3.4.1 Anthropometry

Valid height and weight measurements were obtained for 90% (47/52) of participants at baseline and follow-up. Five children lacked follow-up data due to absence (*n* = 4) or declining participation (*n* = 1).

#### 3.4.2 Accelerometery

At baseline, 75% (*n* = 39/52) provided valid accelerometer data. Invalid measurements resulted from device issues (*n* = 4), refusal (*n* = 2), absence (*n* = 2), or device loss (*n* = 1). Only those with valid baseline data wore accelerometers at follow-up. Of the original sample, 71% (*n* = 35) returned valid follow-up data. Invalid data resulted from absence (*n* = 1), leaving preschool (*n* = 2), or device malfunction (*n* = 5).

#### 3.4.3 Post-intervention questionnaire response

Post-intervention, 75% (3/4) of teachers and 45% (9/20) of parents returned valid surveys. Five parent surveys were incomplete and therefore excluded.

### 3.5 Behavioural and health outcomes

For participants with valid baseline accelerometer data (*n* = 39; intervention *n* = 20, control *n* = 19), the mean daily minutes of total PA were 159.79 ± 44.79 in the intervention group and 161.10 ± 28.23 in the control group. The mean daily steps were 8,965.20 ± 3,030.03 in the intervention group and 8,826.88 ± 3,165.6 in the control group. The intervention group spent 406.33 ± 93.52 min per day sedentary, whilst the control group spent 398.11 ± 71.75 min per day sedentary at baseline (*p* > 0.05). The average time wearing an accelerometer per day was 566.12 ± 68.257 min in the intervention group and 559.22 ± 73.06 min in the control group. There were no significant between-group differences in any PA variables (Table 2).

Whilst the intervention group demonstrated higher total PA minutes at the 10-week follow-up compared to the control group, the changes within and between groups across outcomes were nonsignificant. Specifically, from baseline to 10 weeks, the intervention group increased moderate-to-vigorous PA by 8.01 min/day (95% CI−19.2 to 3.12; *p* = 0.147) whereas the control group decreased by 4.04 min/day (95% CI−6.11 to 14.20; *p* = 0.411). Regarding total daily sedentary time, the intervention group decreased by 18.07 min/day (95% CI−45.5 to 66.9; *p* = 0.694) and the control group increased by 8.9 min/day (95% CI−37.9 to 65.9; *p* = 0.571). Despite more favourable changes in the intervention versus control group for moderate-to-vigorous PA, light activity, and sedentary time, there were no statistically significant within-group changes or between-group differences from baseline to 10 weeks across any outcome measures (Table 2).

## 4 Discussion

This study examined the feasibility of conducting a cluster randomised controlled trial of the IAAH intervention in preschools. The participating preschools were willing to be randomly assigned to study conditions. We assessed the feasibility of recruitment, follow-up, data collection, intervention attendance, and the implementation of school-based and parent-based intervention components. Overall, the findings showed that the intervention was delivered as intended to all participants. Further, it was well-received by both teachers and parents and considered feasible and deliverable. However, the results suggested that some modifications to the study intervention delivery are needed before moving on to the next stage of evaluation.

The recruitment rate at the cluster level in this study (13/112, 12%) demonstrated similarities with a comparable preschool PA intervention (11% uptake) (76), though was lower than what has been observed in other early childhood feasibility studies (77–79). The individual-level recruitment rate (52/143, 36%) was consistent with existing preschool research reporting uptake rates ranging from 25 to 52% (77, 78, 80). Whilst our trial employed comparable recruitment methods to those seen in a previous high-enrolment study (57), the socioeconomic disparity across samples may explain the recruitment discrepancy. Specifically, the prior trial likely benefited from medium-to-high socioeconomic status (SES) preschools, whereas our sample was skewed low-to-middle SES. Extensive literature has documented greater recruitment and retention difficulties amongst economically disadvantaged populations (81, 82). To optimise the future trial recruitment of preschoolers, it is advisable to contemplate diversifying SES and employing tailored methods for hard-to-reach groups. For the planned cluster randomised controlled trial, proactive retention strategies will be used such as reminders, incentives, and closely monitored participation tracking (83) to boost recruitment and minimise attrition.

Overall participant retention in this study surpassed that of comparable studies, reaching 90%, with a minimal 10% attrition rate after the 10-week period. This is in contrast to 68–75% retention observed at 12 weeks in other studies (57, 77, 84). However, amongst those participants who completed follow-up, the amount of valid data collected differed substantially depending on the outcome measure. The noteworthy accomplishment of a 95% attendance rate in this feasibility study exceeded the attendance reported in similar preschool interventions in the UK (53%) (85) and Finland (70.4%) (86). This provides promising indications that the intervention can successfully engage and retain preschool participants. The maintenance of strong attendance will be a pivotal focus as the study advances into the larger-scale effectiveness trial.

The intervention was delivered with high fidelity (93.3%) in the preschool setting. Logbook responses showed that PA components were implemented at a higher level than SB components (Table 2). Previous studies also found relatively low implementation scores for SB activities across multiple sites (36, 87, 88). Considering these findings, despite adapting the programme to reduce time-intensive activities, poorer SB implementation highlights the need for further modifications to the SB components.

However, implementation fidelity for the home component was 74%. This is consistent with other preschool interventions, including home elements (89). These results are unsurprising, as the home environment has been identified as particularly challenging for implementing obesity prevention efforts (85), especially in disadvantaged groups. The low fidelity of the home component highlights the difficulties of extending preschool interventions into the home setting. Strategies to improve engagement and adherence for home-based components should be explored before implementing similar interventions. Overcoming barriers in the home environment is key to maximising the effectiveness of preschool interventions which target healthy lifestyle promotion.

In this study, 90% of participants completed height and weight measurements, aligning with the anthropometric measurement rates achieved in similar studies (90–92), indicating that these procedures are feasible in the preschool population.

Regarding accelerometery, at baseline, 39 children (75%) provided valid data, but only 35 (71%) had valid wear time at both baseline and 10 weeks. This aligns with other preschool RCTs reporting 42–80% valid wear time at both timepoints (i.e., baseline and follow-up) (57, 93, 94). There were several factors that prevented the collection of valid accelerometer data at both timepoints in previous studies, such as device loss or malfunction and child absence on the data collection day (36, 95–97). The literature offers inconsistent findings regarding compliance with accelerometer measurement procedures in studies assessing PA and SB in children. Recent reviews of attrition rates and noncompliance with accelerometer protocols in children’s PA trials indicate that noncompliance at follow-up assessments ranged greatly from 3 to 70% across 23 studies (98).

Previous studies using Actigraph accelerometers for brief 2-day wear periods reported a high adherence rate of 96–97% at baseline and 6-month follow-up (99), suggesting that a shortened monitoring period could improve compliance. However, another study requiring 7 days of Actigraph wear in preschoolers achieved only 86% adherence, indicating that additional factors likely influence accelerometer compliance beyond wear duration alone (100). As the ActiGraph GT3X was comparable to other potentially less invasive and participant-friendly wearable devices (65, 101), there may be valid alternatives which could be considered for future trials. Regardless of the device chosen, enhancing communication with parents throughout recruitment and follow-up could facilitate collecting more valid accelerometer data in any future trial. Multiple studies have shown favourable results by utilising strategies such as reminder messages, check-in calls, and small monetary incentives for accelerometer returns (91).

In this study, only 45% of parents responded to the post-intervention questionnaire, indicating even lower engagement than the suboptimal benchmark. This response rate is comparable to some previous studies (for example, 48%) (88) but lower than others (for example, 75%) (102). Whilst we attempted to shorten the questionnaires before starting the trial, further adaptation may be necessary to improve response rates. Several factors likely contributed to the modest questionnaire return rate in this study. Poor awareness about the importance of the questionnaire and its potential to inform decision-making, as well as privacy concerns, may have reduced participation. Some may have believed that offering opinions would not impact the situation. To optimise response rates in future trials, more efficient questionnaire alternatives should be explored, such as clarifying the purpose of the research, ensuring privacy, simplifying the design, offering small incentives, using direct communication methods like phone or SMS reminders, and providing flexible response options (for example, paper, electronic, or phone). Generally, understanding and accommodating the target community’s needs and expectations when designing the questionnaire may elicit improved engagement and results in subsequent studies (103).

Comparing the findings of this feasibility study with prior research offers valuable insights into the potential impact of the intervention. Although our intervention demonstrated enhancements in step counts, BMI, moderate-to-vigorous PA (MVPA), and SB (SB), these improvements did not reach statistical significance. This concurs with certain studies (85, 86) but diverges from others that reported significant enhancements in comparable outcomes (57, 104). Conversely, some studies identified increases in BMI and inactivity post-intervention, with no discernible changes in PA (76). The absence of statistical significance in our results may be attributed to the brief 10-week duration, as more substantial effects could necessitate interventions lasting 6 months or longer (41). This underscores the imperative for a more comprehensive evaluation in an upcoming randomised controlled trial (RCT), wherein augmented sample sizes and an improved study design should enhance the precision of assessing effectiveness.

Should our intervention prove effective in an RCT, the implications for promoting PA and self-efficacy amongst young children would be significant. Successful interventions documented in other studies have underscored that meticulously planned and supported initiatives can yield positive outcomes (99). Scaling up and integrating our intervention into health and education curricula would align with the success observed in analogous endeavours (34), thereby reinforcing the importance of engagement with key stakeholders.

Furthermore, considering the broader ramifications of PA, our intervention aligns with the idea that fostering healthy behaviour can positively impact other aspects of well-being. This is corroborated by research indicating that PA interventions have favourable effects on behaviours such as diet, sleep, and overall wellbeing (105).

## 5 Strengths and limitations

The main strength of this study was the first systematic pilot testing of the feasibility of implementing the IAAH intervention in Saudi Arabian preschools, filling a significant gap in research into delivering a physical activity promotion programme tailored for young children in this context. Additionally, the multimethod data collection allowed for a comprehensive evaluation of feasibility parameters.

However, this study had some key limitations. The sample was restricted to preschools in one urban area, which limits broader generalizability. The modest sample size, whilst reasonable for a feasibility study, precluded blinding of participants or intervention providers. Several feasibility outcomes, such as questionnaire response rates and accelerometer compliance, were suboptimal and highlighted target areas needing improvement before an effectiveness trial. As this pilot was not powered to detect intervention effects, assessments of preliminary outcomes should be interpreted with caution. Finally, detailed information on the acceptability of the intervention procedures and content from participants and providers would further contextualise the feasibility findings this aspect remains unexplored in the current study.

## 6 Conclusion

This feasibility study has provided critical insights into the implementation of the IAAH intervention within preschools settings, demonstrating promising recruitment and retention rates and indicating that a larger-scale trial is both feasible and warranted.

The feasibility of conducting such interventions in preschool settings, along with their acceptable and implementable nature as perceived by facilitators, lays a solid foundation for future large-scale applications aimed at combating childhood obesity. Insights into barriers and facilitators to intervention implementation provide valuable guidance for improving future interventions, ensuring they are more tailored, attractive, and effective. Furthermore, our study highlights the way forward for subsequent trials, particularly by emphasising the importance of strategic improvements in recruitment and data collection methodologies. These improvements will not only improve the power of future research but will also enhance our understanding of effective strategies for promoting physical activity amongst preschool children.

We recommend that interested researchers, key stakeholders, and policymakers pursue a revised approach to the IAAH intervention that incorporates the successful elements identified in this pilot project with necessary modifications based on the challenges encountered and considering environmental, cultural, and other contextual factors. This strategic intervention development is expected to contribute significantly to ongoing efforts to promote a more active and healthier lifestyle from an early age, ultimately helping to address childhood obesity.

## Data availability statement

The original contributions presented in the study are included in the article/Supplementary material, further inquiries can be directed to the corresponding author.

## Ethics statement

The studies involving humans were approved by Saudi Arabian Ministry of Health’s Research and Studies Department’s IRB Registration Number with KACST, KSA: HAP-02-T-067. The studies were conducted in accordance with the local legislation and institutional requirements. Written informed consent for participation in this study was provided by the participants’ legal guardians/next of kin.

## Author contributions

MA-w: Conceptualization, Data curation, Formal analysis, Investigation, Methodology, Resources, Software, Writing – original draft, Writing – review & editing. MD: Conceptualization, Formal analysis, Investigation, Methodology, Resources, Supervision, Writing – review & editing. AA: Conceptualization, Data curation, Formal analysis, Software, Supervision, Writing – review & editing. NH: Conceptualization, Data curation, Formal analysis, Investigation, Methodology, Resources, Software, Writing – review & editing.
