# Supplementary material for: Pre-school-based behaviour change intervention to increase physical activity levels amongst young children: a feasibility cluster randomised controlled trial
Source: Front Public Health. 2024 May 2;12:1379582. doi: 10.3389/fpubh.2024.1379582 (PMC11096494; doi:10.3389/fpubh.2024.1379582)
Supplement: Supplementary file 1 [file Table_1.DOCX]

**Supplementary File S1:**

**The I’m an Active Hero (IAAH) preschool health programme- parent/guardian feedback form.**

| The questions below ask about the materials that accompanied the I’m an Active Hero programme. Please tick **one** box for each question. | **Yes** | **No** | **Not sure** |
| --- | --- | --- | --- |
| Did you receive posters, tip cards and newsletters information about the I am an Active Hero program from your child's preschool? |  |  |  |
| Did you receive a Family Achievement Sheet (to record physical activity and sedentary behaviours)? |  |  |  |
| Did you receive the home activity pack called “Mirror, Mirror!”? |  |  |  |
| Did you receive the home activity pack called “Freeze Dance”? |  |  |  |
| Did you receive the home activity pack called “Galloping”? |  |  |  |
| Did you receive the home activity pack called “Roll the Dice”? |  |  |  |
| Did you receive the home activity pack called “Animal Friends”? |  |  |  |
| Did you receive the home activity pack called “No TV Day’ challenges”? |  |  |  |

| The next two questions are about your use of the I’m an Active Hero home material. Please tick **one** box for each question | **None** | **Some** | **Not sure** | **Most** | **All** |
| --- | --- | --- | --- | --- | --- |
| How many of the I’m an Active Hero home activities that you received did you use at home with your child? |  |  |  |  |  |
| Have you given your child the opportunity to lead the activities? |  |  |  |  |  |

| The next questions are about your feelings towards the I’m an Active Hero home material. Please tick **one** box for each question | **Strongly disagree** | **disagree** | **Not sure** | **agree** | **Strongly agree** |
| --- | --- | --- | --- | --- | --- |
| Overall, did your child enjoy the activities in the programme? |  |  |  |  |  |
| Did you enjoy doing the activities with your child? |  |  |  |  |  |
| Do you think the activities helped your child be more physically active? |  |  |  |  |  |
| Do you think the activities helped your child spend less time sitting/being inactive? |  |  |  |  |  |
| Were the instructions provided for the games/activities easy to read and clear? |  |  |  |  |  |

Please provide any additional comments that you would like to make about the I’m an Active Hero home materials and activities in the box below:

**Thank you for taking the time to complete this feedback form!**
